# Gut microbiota, circadian rhythms and their interactions: implications for the pathogenesis and treatment of depression

**DOI:** 10.1080/07853890.2025.2561222

**Published:** 2025-09-18

**Authors:** Dongru Du, Yanling Yuan, Xuan Guan, Qinglian Xie, Xueli Sun, Zaiquan Dong

**Affiliations:** ^a^ West China School of Medicine, Sichuan University, Chengdu, People’s Republic of China; ^b^ Department of Pharmacy, West China Hospital, Sichuan University, Chengdu, People’s Republic of China; ^c^ Chengdu Medical College, Chengdu, People’s Republic of China; ^d^ Department of Outpatient, West China Hospital of Sichuan University, Chengdu, People’s Republic of China; ^e^ Department of Outpatient, West China Xiamen Hospital of Sichuan University, Fujian, People’s Republic of China; ^f^ Mental Health Center, West China Hospital, Sichuan University, Chengdu, People’s Republic of China

**Keywords:** Depression, gut microbiota, circadian rhythm, bidirectional interaction, treatment innovation

## Abstract

**Background:**

Depression is a complex and widespread mental illness that remains poorly understood in terms of pathogenesis and treatment. Recent research has highlighted the intricate connections between gut microbiota and circadian rhythms and indicated that they may serve as significant factors associated with depression.

**Discussion:**

The gut microbiota and its metabolites may affect circadian rhythms *via* microbial oscillations, neurotransmitters, vagus nerve, epigenetic modifications and immune regulation, whereas the circadian rhythms may affect the gut microbiota *via* circadian gene expression, neurotransmitters, vagus nerve and immune regulation. These findings may provide new perspectives on the pathogenesis of depression and have led to the development of novel treatments targeting the gut microbiota, circadian rhythms and their interactions. This review explores how disturbances in the gut microbiota and circadian rhythms may contribute to depression and discusses the innovative treatments that have resulted from this knowledge.

**Conclusion:**

By focusing on this emerging area of study, this review reveals promising new developments in the pathogenesis and treatment of depression, emphasizing the novel concept that balancing our internal microbiome and body clock could be key to effectively managing this condition.

## 1. Introduction

Depression is a prevalent mental disorder characterized by persistent loss of interest in life and sadness [1]. A nationwide epidemiological study reported that the lifetime prevalence of depression in China has reached 6.8% [2]. Despite recent advances in the identification of potential therapeutic targets of depression, the underlying mechanisms of which remained unclear [3,4].

Recent evidence suggests that gut microbiota may serve as a crucial indicator that could help elucidate the pathogenesis of depression [5]. The gut microbiota encompasses diverse and dynamic microorganisms within the human intestine, which significantly impact various aspects of human health, including immune regulation [6], energy metabolism [7] and cognitive performance [8]. Interactions between the gut microbiota and central nervous system (CNS) have been extensively studied and are commonly summarized as the microbiota-gut-brain (MGB) axis [9]. The gut microbiota influences CNS function through multiple pathways, including metabolite secretion, neurotransmitters, vagus nerve stimulation and immune modulation [9]. Factors such as antibiotics, infections and stress responses can alter the abundance and function of the gut microbiota, thereby affecting CNS function and increasing the risk of mental disorders [10]. New microbial-related pathways have also been identified to explore the pathogenesis of mental disorders. We previously published a review discussing the impacts of the gut microbiota on CNS disorders *via* epigenetic mechanisms, including DNA methylation, histone modification, and the action of non-coding RNAs [11]. Another study suggested that host metabolism mediates the relationship between gut microbiota remodelling and depressive-like behaviours [12]. Thus, exploring new pathways by which the gut microbiota could influence depression may provide a more comprehensive understanding of its pathogenesis.

To adapt to the 24-hour dark-light cycle caused by Earth’s rotation, humans and animals have developed circadian rhythms, which are crucial for optimizing the timing of various physiological processes and behaviours [13]. Disruption of the circadian rhythm is associated with an increased risk of several mental disorders, including delirium, dementia and depression [14]. A prospective observational study suggested a close relationship between circadian phase disturbances and the severity of mood symptoms in patients with depression [15]. Another study found that circadian disruption impaired oligodendrocyte myelination *via* Bmal1 overexpression, leading to depression-like behaviours [16]. These results indicate that the circadian system may also play a role in the pathogenesis of depression.

Given that both the gut microbiota and circadian rhythms are significant factors potentially correlated with depression, their intricate connections have become a hot topic in recent studies. These connections may offer a novel perspective on the pathogenesis of depression [17]. The oscillations and metabolism of the gut microbiome may influence host circadian rhythm function, affecting the coordination of various host systems, whereas circadian rhythm regulation regulates microbial rhythmicity and microbiome-body synchronization [13,18]. In addition, another study found that acute sleep deprivation and circadian rhythm disruption can exacerbate psychiatric disorders through gut microbiota dysbiosis [19,20]. However, the effects of gut microbiota-circadian rhythm interactions on the pathogenesis and treatment of depression are not yet fully understood. In this review, we summarize the potential roles of gut microbiota, circadian rhythms, and their interactions in the pathogenesis and treatment of depression, offering new insights into the management of depression and related mental disorders.

## 2. Gut microbiota

### 2.1. Factors affecting the composition and function of the gut microbiota

Both intrinsic and extrinsic factors can influence the composition and function of the gut microbiota. Intrinsic factors such as genetics and age play crucial roles in shaping and maintaining the microbiota [21]. Extrinsic factors, including diet, exercise, drugs and the light-dark cycle, may affect the composition and function of the microbiota. For instance, microbes such as *bifidobacteria* and *lactobacilli* significantly increase in response to fibre-rich diets. Conversely, individuals receiving fibre-free synthetic enteral nutrition were found to have increased abundance of *Ruminococcus gnavus* and *Ruminococcus torques* [22,23]. A well-balanced diet, like the Mediterranean diet, maintains the function of epithelial barrier by promoting the production and effects of short-chain fatty acids (SCFAs). In contrast, an unbalanced diet, such as the western diet, can decrease SCFAs production and impair epithelial barrier function [24]. Regular exercise has also been shown to stabilize the intestinal microecological environment [25]. In obese children, exercise has been shown to shift the microbiota profile towards that of healthy children by reducing the abundance of *Gammaproteobacteria* and *Proteobacteria* and increasing the abundance of *Blautia*, *Dialister* and *Roseburia* [26]. Conversely, sedentary behaviour is linked to increased *Escherichia coli* abundance, which decreases with increased physical activity [27]. Interactions between various drugs and the gut microbiota are also well-documented. The use of antibiotics, particularly broad-spectrum ones, is associated with reduced alpha diversity and incomplete recovery of the microbiome in adults [28]. Other medications, such as antidepressants, may influence the treatment of depression by altering the composition and abundance of the microbiota [9]. In addition, modern lifestyle factors such as international travel and shift work can affect the gut microbiota *via* disruptions of the light-dark cycle [25]. International travel is linked to decreased sleep efficiency, which also impacts the composition and function of the gut microbiota [19,29]. Shift work, characterized by nighttime sleep deprivation, disrupts the healthy functioning of the gut microbiome and increases the risk of multiple diseases [30,31].

### 2.2. Effect of gut microbiota dysbiosis on the pathogenesis of depression

Gut microbiota dysbiosis can affect the pathogenesis of depression *via* multiple pathways, including microbiota-derived metabolites, neurotransmitters, the vagus nerve and immune regulation [9].

Microbiota-derived metabolites, such as SCFAs, participate in the pathogenesis of depression [32]. Preclinical and clinical evidence suggests that SCFA levels decreased in patients with depression, whereas rifaximin and inulin may reduce depressive symptoms by upregulating SCFA levels [20,33]. Mechanically, Zhang et al. reported that microbiota-derived SCFAs may alleviate depressive behaviours in mice by upregulating the Sigma-1 receptor/brain-derived neurotrophic factor/tyrosine kinase pathway [34].

Neurotransmitters such as serotonin may also be affected by microbiota dysbiosis and participate in the pathogenesis of depression. Gut microbiota dysbiosis may disturb serotonergic neurotransmission by affecting the availability of tryptophan, thereby increasing the risk of depression [35]. González-Arias et al. reported that mice in a depressive-like state exhibit reduced astrocytic Ca^2+^ signalling driven by serotonin, while upregulation of astrocytic Ca^2+^ signalling reduces behavioural deficits in mice [36]. Tian et al. suggested that *Bifidobacterium breve* CCFM1025 (a novel probiotics) may attenuate depression by modulating gut microbiota and tryptophan metabolism [37].

The vagus nerve directly identifies signals from the gut microbiota with the help of toll-like receptors on its membrane surface, providing a direct pathway linking the gut microbiota and CNS [38]. Decreased vagal tone may promote systematic inflammation and gut microbe translocation, thereby increasing the risk of depression [39].

Finally, immune-related mechanisms also exert significant effects, linking gut microbiota dysbiosis and depression. Normally, the interplay between the gut microbiota and intestinal epithelium maintains homeostasis and prevents excessive inflammation [40]. Disruptions in the gut microbiota are associated with increased gut permeability and exposure to abnormal bacterial lipopolysaccharides, which may increase the levels of pro-inflammatory cytokines and abnormal glial functions, contributing to the pathogenesis of depression [41].

Summary: The composition and function of the gut microbiota can be shaped by multiple intrinsic or extrinsic factors. Gut microbiota dysbiosis may contribute to depression *via* microbial-related metabolites, neurotransmitters, the vagus nerve and immune regulation.

## 3. Circadian rhythm

### 3.1. Circadian rhythm and its molecular mechanisms

The circadian rhythm is regulated by a central pacemaker and multiple peripheral oscillators [42]. The central pacemaker, located in the suprachiasmatic nucleus (SCN) of the anterior hypothalamus, comprises nearly 10,000 neurons [42]. Each neuron possesses a cell-autonomous circadian oscillator that coordinates circadian clock activity in peripheral tissues [43]. Peripheral oscillators govern a wide range of cellular and molecular processes, including the transcription and regulation of cell physiology [43].

**Figure 1.**
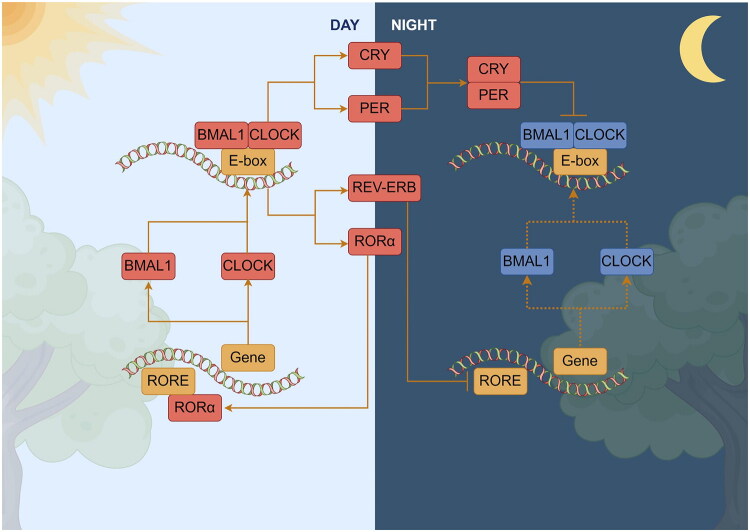
Molecular mechanisms of the circadian system, created by Figdraw. Clock: circadian locomotor output cycles kaput; BMAL1: brain and muscle Arnt-like protein 1; CRY: cryptochromes; per: period; and RORE: retinoic acid orphan receptor element.

The maintenance of circadian rhythms relies on interconnected negative feedback and transcription-translation loops (Figure 1) [44]. In the core loop, the heterodimeric relationship between circadian locomotor output cycle kaput (CLOCK) and brain and muscle Arnt-like protein 1 (BMAL1) acts as the activator [45]. CLOCK and BMAL1 form a heterodimer in the cytoplasm, which then enters the nucleus to bind to the Enhancer Box (E-box) and activate the expression of cryptochromes (CRY1 and CRY2) and periods (PER1, PER2, PER3). CRY and PER then form the CRY-PER complex in the cytoplasm and re-enter the nucleus, inhibiting the CLOCK-BMAL1 complex [46]. CRY and PER levels are regulated by specific E3 ubiquitin ligase complexes that control polyubiquitination and degradation rates [21]. In addition, essential regulators such as nuclear receptor subfamily 1 group D (REV-ERB) and retinoic acid orphan receptor element (RORE) form another negative feedback loop. REV-ERBs inhibit BMAL1 expression by disrupting transcription, while RORs upregulate BMAL1 expression by binding to the RORE within their promoters [46].

### 3.2. Factors affecting circadian rhythms

#### 3.2.1. Factors affecting the Central pacemaker

Circadian rhythms are influenced by various external factors known as zeitgebers [21]. The central pacemaker is primarily affected by the 24-hour light-dark cycle [25]. Light signals received by the retina are converted into electrical signals by retinal ganglion cells and are transmitted to the SCN *via* the retinohypothalamic tract [46]. Output mediators, including hormones, metabolites and cytokines, are generated to synchronize peripheral clocks and adapt to the light-dark cycle [47]. Xu et al. found that NPAS4, whose expression is induced by light, can alter the transcriptional response of the SCN to light and regulate circadian behaviour [48]. Joye et al. observed that SCN function is modulated by somatostatin deficiency in a sex-specific manner through mechanisms such as network aftereffects, intercellular synchrony and photoperiodic encoding [49].

#### 3.2.2. Factors affecting peripheral clocks

In addition to central pacemakers, various other factors modulate peripheral clocks of humans, including diet, temperature, exercise and pharmacological treatments [21]. Interactions between diet and circadian rhythms are increasingly recognized as crucial for maintaining physiological activities [50]. Increased clock gene expression and disrupted feeding activity were observed in the adipose tissue of mice fed a high-fat diet, highlighting the importance of a balanced diet for circadian rhythm homeostasis [51]. Environmental temperature also affects peripheral clock regulation. Rabearivony et al. found that mice housed at 26 °C exhibited altered circadian gene expression compared with mice housed at 21 °C [52]. Li et al. suggested that heat exposure increases BMAL1 levels and decreases CLOCK protein levels in testes [53]. Exercise also influences the peripheral clock system. Casanova-Vallve et al. reported that daily exercise improves circadian rhythms in skeletal muscles, affecting both their physiological functions and molecular characteristics [54]. Another study on sedentary adults found that timed exercise caused phase shifts in the circadian rhythm in a chronotype-dependent manner, indicating that personalized exercise prescriptions could potentially be used to address circadian misalignment [55]. In addition, pharmacological treatments can affect circadian rhythms. He et al. demonstrated that Sini powder (a traditional Chinese formula) and paroxetine alleviate depression through circadian rhythm modulation [56]. A systematic review indicated that typical antipsychotics (such as flupentixol and haloperidol) can disrupt daily rhythms, whereas atypical antipsychotics (such as risperidone and clozapine) can restore these rhythms [57].

#### 3.2.3. The role of vagus nerve in circadian rhythm

Previous studies have supported a link between vagus nerve and circadian rhythm. Chrobok et al. reported that the dorsal vagal complex of brainstem was regulated by internal circadian rhythm, which may affect its genetic and vascular activities [58]. Wang et al. reported that stimulation of auricular vagus nerve may improve the concentration of melatonin and inhibit thermal hypersensitivity in rats [59]. Further evidence suggested that the vagus nerve may also serve as a bridge linking the central pacemaker and peripheral clocks together. Woodie et al. reported that in mice with liver circadian clock dysfunction, signals were delivered to the brain *via* hepatic vagus nerve, leading to an abnormal food intake pattern [60]. Future studies may focus on the role of vagus nerve between the central pacemaker and other peripheral clocks.

### 3.3. Effect of disrupted circadian rhythm on depression

Disrupted circadian rhythms may affect the pathogenesis of depression. Accumulating evidence suggests that this process may involve multiple pathways, including light exposure, the hypothalamic-pituitary-adrenal (HPA) axis, neurogenesis and monoamine signalling [61].

Abnormal light exposure is an important cause of circadian disruption and it contributes to the pathogenesis of depression. Since the advent of electronic lighting, humans have been exposed to excessive artificial light in public areas and at home, which may disrupt internal circadian rhythms and increase the risk of mood disorders [62,63]. Lei et al. also indicated that circadian disruption caused by artificial light may lead to cognitive and emotional impairment through neuroinflammation and oxidative stress [64]. Zuo et al. found that mice exposed to long-term variable photoperiods experienced circadian misalignment, which disrupted oligodendrocyte myelination and led to depression and anxiety-like behaviours [16].

The HPA axis is closely connected to circadian systems and stress responses [65]. The central pacemaker in the SCN controls the release of corticotropin releasing hormone (CRH) from the hypothalamic paraventricular nucleus, which then stimulates the release of adrenocorticotrophic hormones (ACTH). ACTH are responsible for stimulating the release of glucocorticoids, which exert negative feedback on CRH and ACTH [65]. Increasing evidence suggests that the HPA axis is a crucial pathway through which circadian rhythm disruption contributes to the pathogenesis of depression [61]. The destruction of the SCN can disrupt the secretion of HPA-related hormones, thereby alleviating depressive-like symptoms in animal models. Conversely, Cry2-knockout mice are associated with increased corticosterone levels, which may exacerbate depression-like and anhedonic behaviours [61,66,67].

Neurogenesis is a process by which precursors generate functional neurons in specific regions, which play essential roles in the activity and functioning of the CNS [68]. Circadian rhythm disruption can inhibit neurogenesis in the hippocampus, which is consistent with the results in patients with depression [61,69]. However, evidence supporting the idea that disruptions to circadian rhythms contribute to depression *via* neurogenesis inhibition remains inadequate.

Finally, monoamine signalling may also serve as a crucial pathway through which circadian rhythm disruption contributes to the pathogenesis of depression. SCN destruction has been shown to disrupt the expression of tyrosine hydroxylase and dopamine transporters, which participate in the pathogenesis of depression by affecting dopaminergic transmission [70]. Serotonin, a key monoamine in mood regulation, interacts bidirectionally with the circadian system [71,72]. The regular output of the SCN modulates the function of the serotonin system, whereas serotonin neurons project back to the SCN to regulate circadian rhythms [73]. Disruptions in circadian rhythms may also exacerbate depressive symptoms by affecting the serotonin innervation of mood-related regions in the CNS [72].

Summary: Circadian rhythms are affected by multiple intrinsic and extrinsic factors similar to the gut microbiota. Light exposure, the HPA axis, neurogenesis and monoamine signalling may participate in the process of which disrupted circadian rhythm contribute to the pathogenesis of depression.

## 4. Interactions between the gut microbiota and circadian rhythms

### 4.1. Effects of the gut microbiota on circadian rhythms

Previous studies have demonstrated the regulatory effects of gut microbiota on the circadian rhythms. Wang et al. showed that microbiota-induced expression of the epithelial transcription factor NFIL3 occurs *via* the DC-ILC3 signalling and REV-ERBα thereby modulating various body functions [74]. Kuang et al. emphasized the role of gut microbiota in the programming of diurnal rhythms through histone deacetylase 3 [75]. Studies reporting the role of gut microbiota in circadian syndrome was summarized in Table 1 [74–82]. The gut microbiota and its metabolites may modulate circadian rhythms through multiple pathways, including microbial oscillations, neurotransmitters, vagus nerve stimulation, epigenetic modifications and immune regulation [83].

**Table 1.** Studies reporting the role of gut microbiota in circadian rhythm.

Author year	Species	Methods	Conclusion
Tofani 2025 [73]	C57BL/6J mice	Gut microbiota depletion, acute restraint stress, faecal microbiota transfer, blood brain barrier assessment and metabolomics.	Microbiota regulates stress responsiveness in a circadian manner
Zhang 2023 [74]	Drosophila	Timed feeding and gut microbiota depletion.	Microbiome stabilized circadian rhythm in the host gut.
Schugar 2022 [75]	C57Bl6/J and *Lep^ob/ob^* mice	High-fat diet and untargeted metabolomics.	Trimethylamine derived from gut microbiota is a key regulator of the host circadian clock
Leone 2022 [76]	Male C57Bl6/J mice (Conventional or germ-free)	Normal or high-fat diet.	Microbes affected the interactions among food- and light- entrainable circadian rhythm.
Huang 2022 [77]	BALB/c female germ-free mice	Faecal microbiota transplantation, transcriptomic and metabolomics	Seasonal changes of gut microbiota may synchronize host peripheral circadian rhythms and regulate physiological adaptation.
Fawad 2022 [78]	Schaedler Flora and C57Bl/6J mice	Organoid approaches and systems metabolomics.	SCFAs regulated intestinal epithelial circadian rhythms by an HDACi-dependent mechanism.
Brooks 2021 [79]	Wild-type C57BL/6, Reg3g^−/−^, Myd88^−/−^, Myd88^ΔIEC^, Myd88^ΔDC^, Stat3^ΔIEC^, Rag1^−/−^, Rorc^gfp/gfp^, Stat3^ΔIEC^, Nr1d1 ^−/−^ (Rev-erbα-deficient) and Clock ^Δ19/19^ mice	Bacterial infection and segmented filamentous bacteria monocolonization.	The microbiota and circadian clock regulate the diurnal rhythms of innate immunity
Kuang 2019 [72]	Wild-type C57BL/6, Hdac3^fl/fl^, Hdac3^ΔIEC^, Nr1d1^–/–^ (REV-ERBa-deficient) and Myd88^–/–^ mice	Jet lag experiments, high-fat vs regular chow diet.	Gut microbiota regulated circadian rhythm of host *via* HDAC3
Wang 2017 [71]	C57BL/6 wild-type, *Nfil3*^Δ^*^IEC^, Rev-erb*α*^–/–^, Cd11c-*DTR*, Myd88^–/–^*, *Myd88*^Δ^*^IEC^*, *Myd88*^Δ^*^DC^*, *Rag1^–/–^*, *Rorc^gfp/gfp^* and *Stat3*^Δ^*^IEC^* mice	High-fat vs regular chow diet.	Gut microbiota regulated body composition *via* NFIL3 and the circadian rhythm.

The gut microbiota exhibits 24-hour oscillations that are promoted by a low-fat, fibre-rich diet and reduced by a high-fat, fibre-free diet [83]. These oscillations influence the circadian system by affecting the transcriptional and epigenetic regulation of circadian rhythms [84] and by aligning dietary cues with the host circadian network [13]. In addition, microbial metabolites such as SCFAs and bile acids modulate circadian rhythms [85]. Tahara et al. found that SCFAs derived from gut microbiota entrain circadian clocks in the peripheral tissues of mice [86]. SCFAs promote serotonin secretion by stimulating tryptophan hydroxylase 1, and serotonin modulates the effects of light and promotes non-photic phase shifts to regulate the circadian system [35,72]. SCFAs also enhance vagus nerve function independently of cholecystokinin [87,88]. In terms of epigenetic regulation, evidence suggests that gut microbiota and their metabolites affect the circadian rhythms through histone modifications [89]. For instance, SCFAs regulate HDAC3 in intestinal epithelial cells, influencing the rhythmicity of H3K9ac and H3K27ac, thereby affecting the circadian rhythms related to lipid metabolism and nutrient uptake [81,89]. The gut microbiota and its metabolites also coordinate interactions between the circadian system and immune cells [90]. SCFAs influence immune cells and modulate inflammatory responses, which in turn upregulate Bmal1a, Clock1b and Per1a in the zeitgeber cycle [4,91,92]. In addition, the increased expression of peripheral Cry2, Per1, Per2 and Per3 in mice treated with unconjugated bile acids suggests that these acids may also affect circadian rhythms [93].

### 4.2. Effects of circadian rhythms on gut microbiota

Some studies have also identified the effects of circadian rhythms on gut microbiota. Wu et al. found that circadian rhythm disorders induced by oral glucocorticoid administration can alter gut microbiota in mice [94]. Amara et al. showed that circadian rhythm disruption intensifies gut microbiota dysbiosis in mice treated with dextran sulphate sodium [95]. Several pathways through which the circadian system regulates the gut microbiota have been identified, including clock gene expression, neurotransmitters, the vagus nerve and immune regulation.

Circadian gene expression temporally regulates the functions of multiple organs and systems [96]. Heddes et al. showed that the circadian system generates diurnal rhythms in the gut microbiota, which can be disrupted by cell-specific Bmal1 ablation [97]. Zhen et al. found that Per2 knockout mice exhibited changes in gut microbiota diversity and reduced SCFA levels, highlighting the crucial role of Per2 in circadian regulation [96,98]. Neurotransmitters such as melatonin also play a significant role in circadian rhythms [99]. Melatonin levels exhibit chronotype-dependent variations and can restore gut microbiota oscillations and increase oscillator abundance in sleep-restricted mice [100,101]. Peripheral clocks in the circadian system may regulate vagus nerve function, thereby influencing gut microbiota abundance [102,103]. In addition, the circadian system controls the storage and release of pro-inflammatory cytokines in immune cells [45]. The disruption of circadian rhythms can exacerbate inflammatory responses, increase intestinal epithelial permeability and affect gut microbiota function [46,104]. Although clock gene expression affects histone acetylation in mice, the effects of circadian rhythms on the gut microbiota in humans through epigenetic modulation require further investigation [105].

Summary: The gut microbiota and its metabolites can influence the circadian system through microbial oscillations, neurotransmitters, the vagus nerve, epigenetic modifications and immune regulation, whereas the circadian system can affect the gut microbiota *via* circadian gene expression, neurotransmitters, the vagus nerve and immune regulation.

## 5. Gut microbiota-circadian rhythm interactions during the pathogenesis of depression

**Figure 2.**
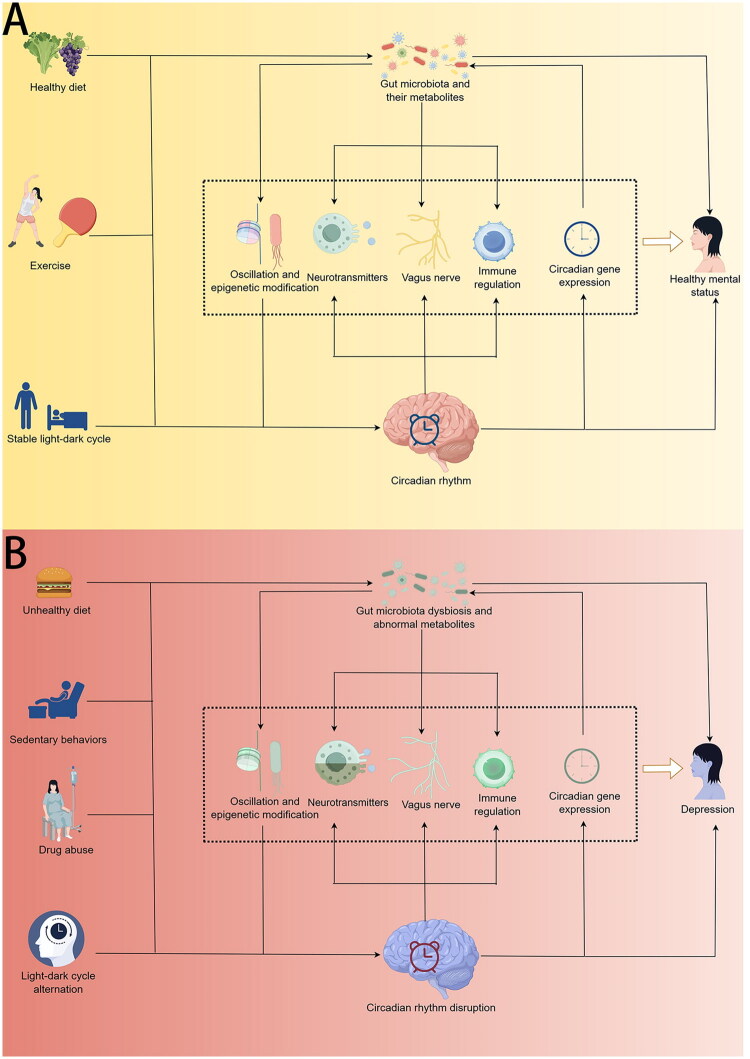
Interactions between gut microbiota and circadian rhythm, created by Figdraw. (a) Health-related determinants, including healthy diet, exercise and a stable light-dark cycle modulate gut microbiota (and their metabolites), circadian rhythm, and their interactions, thereby contributing to the healthy mental status of the host. (b) Factors including unhealthy diet, sedentary behaviours, drug abuse and light-dark cycle alternation may increase the risk of gut microbiota dysbiosis and circadian rhythm disruption, thereby leading to the development of depression. Interactions between disturbed gut microbiota and disrupted circadian rhythms may contribute to the pathogenesis of depression.

Based on the shared influencing factors, similar regulatory effects for mental health and the multiple pathways of interactions between gut microbiota and circadian rhythms, a deeper understanding of gut microbiota, circadian rhythm and their interactions can be achieved. Healthy diet, regular exercise and a stable light-dark cycle are expected to help maintain normal microbiota-circadian interactions and support mental health (Figure 2a), whereas poor diet, sedentary lifestyle, drug abuse and disruptions in the light-dark cycle can lead to gut dysbiosis and circadian rhythm disturbances, which may jointly contribute to the development of depression (Figure 2b). Potential pathways of interplay between the gut microbiota and circadian rhythms that may affect the pathogenesis of depression are summarized below.

### 5.1. Neurotransmitters

Neurotransmitters, including serotonin and melatonin, are crucial for linking circadian rhythms, gut microbiota and depression [35,72,106]. Serotonin, a biogenic monoamine implicated in the pathophysiology of depression, is influenced by both gut microbiota and the circadian rhythms [107]. Zhou et al. found that the abundance of *Roseburia* was lower in patients with MDD than in healthy controls, and *Roseburia* may increase serotonin levels by enhancing tryptophan hydroxylase expression [108]. Rachel et al. noted that serotonin levels are regulated by the circadian system and are influenced by sympathetic nerve signals [72]. For melatonin, several pathways have been revealed to explore its potential antidepressant effects. Ali et al. showed that melatonin reduces pro-inflammatory cytokine levels and alleviates depressive symptoms by regulating FOXO3a and autophagy [109]. Arioz et al. showed that melatonin alleviates depressive symptoms and inhibits NLRP3 inflammasome activation through the SIRT1/Nrf2 pathway [110]. Melatonin is regulated by the circadian system and can help with timing and sleep-wake cycles [111]. In addition, the gut microbiota may influence the regulation of melatonin, and microbiota-derived metabolites, such as butyrate, may mediate the effects of melatonin when treating mental disorders [112].

### 5.2. Vagus nerve

The vagus nerve links the gut microbiota to the CNS and regulates the circadian system, indicating that it plays a role in the pathogenesis of depression [83,113]. Afferent vagal fibres transmit microbial signals to the CNS, whereas efferent fibres mediate inflammation in response to afferent signals [39]. Siopi et al. highlighted the importance of vagus nerve integrity during the development of depression-like behaviours in mice subjected to chronic stress and microbiota inoculation [114]. Yang et al. found that Chrna7 knockout mice displayed depressive behaviours due to disruptions in the MGB axis *via* the subdiaphragmatic vagus nerve [115]. Décarie-Spain et al. demonstrated that the vagus nerve serves as a conduit between peripheral inflammatory responses and the CNS, thereby influencing the development of depression-like symptoms [116].

### 5.3. Epigenetic modifications

Epigenetic mechanisms, including DNA methylation, histone modification, and non-coding RNAs, represent another pathway through which interactions between gut microbiota and circadian rhythms may contribute to depression [11]. Microbiota-derived SCFAs has been shown to inhibit HDACs, thereby promoting histone modifications, including acetylation and crotonylation [117,118]. Clock gene expression was also shown to regulate histone modification in mice [105]. Histone modifications, such as acetylation and crotonylation, have been implicated in pathogenesis of depression [119]. Baek et al. found that an HDAC11 inhibitor alleviates depressive symptoms by inhibiting microglial activation [120]. Liu et al. reported that histone crotonylation, mediated by chromodomain Y-like protein, plays a critical role in regulating depressive symptoms [121]. Further research is required to explore the combined effects of gut microbiota and circadian rhythms on other epigenetic mechanisms that may also contribute to depression [119,122].

### 5.4. Immune regulation

The combined effects of gut microbiota and circadian rhythms may contribute to depression through various immune pathways, including alterations to the T helper 17 (Th17) cells [3]. *Eggerthella lenta* can activate Th17 cells in an antigen-independent manner, whereas the circadian system regulates intestinal immune responses mediated by group 3 innate lymphoid cells/Th17 pathways [123,124]. Th17 cells were also implicated in the development of depression. A meta-analysis revealed higher leukocyte counts and a higher Th17/T regulatory cell ratio in the serum of patients with depression than healthy controls [125]. Peng et al. found that recruiting peripheral Th17 cells into the brain is a critical step in depression, with elevated Th17-derived cytokine levels impairing blood-brain barrier integrity and exacerbating inflammatory responses [126]. Medina-Rodriguez et al. reported that mice lacking Th17 cells were resistant to behavioural changes induced by microbiome alterations in depressed individuals [127]. Further in-depth investigations are required to better understand other immune pathways involved in these interactions.

Summary and concerns: Abnormal interactions between gut microbiota and circadian rhythms may promote the pathogenesis of depression *via* multiple pathways, including neurotransmitters, vagus nerve, epigenetic modifications and immune regulation. However, despite theoretical support, there is limited direct evidence highlighting the impact of this interaction in relation to depression. Future studies should further investigate these pathways to identify direct links between gut microbiota-circadian rhythm interactions and depression.

## 6. Therapeutic potential of gut microbiota, circadian rhythm and their interactions in depression

**Figure 3.**
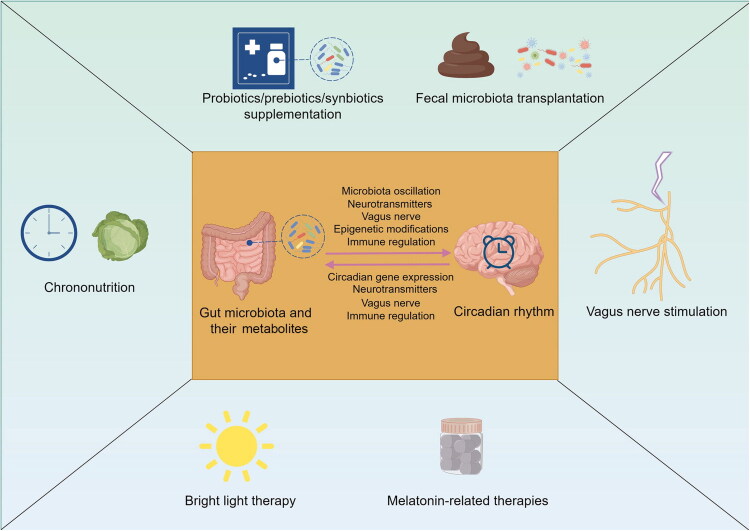
Potential therapeutic approaches for depression considering the impacts of gut microbiota, circadian rhythm and their interactions, created by Figdraw.

The exploration of the intricate connections between gut microbiota and circadian systems has led to the development of novel therapeutic strategies targeting both components and their interactions (Figure 3).

### 6.1. Probiotic/prebiotic/synbiotic supplementations and faecal microbiota transplantation

Given the widespread observation of gut microbiota dysbiosis in patients with depression, modifying or reconstructing the gut microbiome may be a potential therapeutic strategy. This can be achieved with the use of probiotic, prebiotic, and synbiotic supplementations, and Faecal microbiota transplantation (FMT) [21,128].

Probiotics are beneficial microbiomes that can alter the gut microbiota thereby inhibiting the inflammatory response and improving depressive symptoms [129,130]. In a placebo-controlled double-blind trial, Nikolova et al. showed the promising acceptability and tolerability of probiotics in patients with depression [131]. Yamanbaeva et al. found that probiotics altered resting-state functional connectivity in the frontal limb and prevented neuronal degeneration in the uncinate fasciculus [132]. Tian et al. reported that multi-probiotic treatments produced antidepressant-like effects and enhanced serotonin levels in the brainstem and prefrontal cortex of stressed mice, indicating that probiotics may alleviate depressive symptoms by modulating the serotonergic system [133]. Prebiotics, which are selectively fermented dietary components, can regulate abundance and activity of gut microbiota thereby exerting beneficial effects [129]. Chung et al. showed that a mixture of long-chain fructooligosaccharides and short-chain galactooligosaccharides increased SCFA levels and promoted tight junction-related gene expression, potentially alleviating depressive symptoms in sleep-deprived mice [134]. Dietary patterns and administration timing can influence the effects of inulin on stress-induced depressive symptoms in mice [135]. Synbiotics, which combine probiotics and prebiotics, offer a new approach to gut microbiota modulation [129]. Zhang et al. found that synbiotics were more effective against depressive symptoms than single prebiotics [136] or probiotics [137]. Moreover, microbial-related supplements are associated with better sleep quality and circadian homeostasis, which helps reduce depressive symptoms [138,139]. In addition, microbial-related supplements are linked to improved sleep quality and circadian homeostasis, which may help reduce depressive symptoms [140]. Future research should focus on optimizing synbiotic administration routes and elucidating specific mechanisms for alleviating depressive symptoms.

FMT involves transferring minimally processed faeces from healthy donors to patients with microbiota-related disorders [141]. Given the widespread microbiome dysbiosis observed in patients with depression, FMT represents a potential therapeutic approach that can reshape the abundance and function of gut microbiota [142]. Preclinical studies have shown the antidepressant effects of FMT in rats, with changes in neurotransmitters and cytokine levels in the hippocampus [143,144]. Rao et al. suggested that the antidepressant effects of FMT may be linked to glial cell inhibition and NLRP3 inflammasome suppression [145]. Jiao et al. reported that microbiota reconstitution *via* FMT improved age-related circadian dysfunction in C57BL/6J mice [146]. However, in human studies, although some case reports have supported the antidepressant effects of FMT, more well-designed randomized controlled trials are needed [147,148].

### 6.2. Bright light therapy and melatonin-related therapies

Bright light therapy (BLT) is an adjunct treatment that can reduce the severity of symptoms of various types of depression, including MDD, non-seasonal depression and perinatal depression [149–152]. BLT has also been shown to improve the sleep quality of patients with depression [153]. Chen et al. found that BLT increased functional connectivity between the frontal cortex and the midbrain in patients with subthreshold depression [154]. Mechanistically, BLT regulates the phase-shifting and synchronizing effects of circadian rhythm disruption, which may alleviate depressive symptoms [155]. Huang et al. demonstrated that BLT reduces depressive symptoms by activating the retinal-ventral lateral geniculate nucleus/intergeniculate leaflet-lateral habenula (retinal-vlGN/IGL-LHb) pathway [156]. In addition, Chen et al. reported that photobiomodulation therapy modulated gut microbiota diversity in mice with Alzheimer’s disease, suggesting a potential treatment pathway for depression [157].

Melatonin-related therapies, including melatonergic agonists and melatonin supplementation, also have potential antidepressant effects. Melatonergic agonists, such as agomelatine and ramelteon, may modulate depressive symptoms by influencing the circadian system and altering neurotrophic factors and cytokines [158,159]. Arango et al. demonstrated that 25 mg/day agomelatine was effective and safe for treating adolescent patients with MDD [160]. Agomelatine has also been shown to improve sleep quality, anhedonia, sexual dysfunction and somatic symptoms in patients with MDD [161,162]. Mechanistically, Lan et al. found that agomelatine alleviated depressive behaviours and neural injury in mice by suppressing the G alpha (2)-protein kinase A-apoptosis signal-regulating kinase 1 pathway [163]. Diez-Echave et al. reported that agomelatine reduced inflammatory responses and restored butyrate-producing bacteria abundance, potentially mitigating depressive symptoms [164,165]. However, while preclinical studies support the antidepressant effects of melatonin, evidence for the efficacy of exogenous melatonin in humans is limited, and thus further research is required [166,167].

### 6.3. Chrononutrition

Diet, an essential determinant of the human gut microbiota, may also modulates circadian rhythms *via* meal timing and nutrients, which is known as chrononutrition [168]. Meal timing and nutrient intake can affect the development of depression [169]. A meta-analysis found that intermittent feeding has a moderately positive antidepressant effect [170]. Preclinical studies support the antidepressant effects and changes in MGB axis parameters associated with time-restricted feeding [171,172]. Regarding nutrient intake, a meta-analysis indicated that the consumption of fish, coffee and dietary zinc were inversely associated with depression incidences [173]. In addition, compounds such as capsaicin, tea polyphenols, apple polyphenols and flavonoids may help to regulate gut microbiota-circadian interactions and prevent depression [174–177].

### 6.4. Vagus nerve stimulation (VNS)

The vagus nerve, which plays a crucial role in the interactions between gut microbiota and circadian rhythms and contributes to depression pathogenesis, is also a valuable therapeutic target for depression [83,113]. VNS encompasses various methods of electrically stimulating the vagus nerve, leading to functional changes and therapeutic responses [178]. Bottomley et al. found that VNS, when combined with standard treatments, provides consistent benefits for patients with treatment-resistant depression [179]. Tan et al. reported that transcutaneous auricular VNS has antidepressant effects similar to traditional antidepressants but with fewer adverse effects [180].

VNS has also shown to be effective at treating specific types of depression, such as post-stroke depression and MDD with peripartum onset [181,182]. Wang et al. identified the α7nAchR/NF-κB pathway as a key mechanism through which VNS exerts its antidepressant effects [183]. Rosso et al. noted that there were neurotrophin alterations in patients receiving VNS; however, further research is required to determine if these changes contribute to the antidepressant effects of VNS [184].

Summary and concerns to be solved: The novel treatments for depression targeting gut microbiota, circadian rhythm and their interactions include probiotic/prebiotic/synbiotic supplementations, FMT, BLT, melatonin-related therapies, chrononutrition and VNS. Treatments targeting gut microbiota may exert potential positive effects in patients with depression and disrupted circadian rhythms, whereas treatments targeting circadian rhythm disruptions may restore gut microbiota from dysbiosis in patients with depression. However, while the aforementioned approaches shed light on novel treatments for depression, they do not benefit all patients equally. This variability may result from the limited effectiveness of individual interventions on gut microbiota-circadian rhythm interactions. Combining multiple therapeutic strategies may improve outcomes for patients with suboptimal responses to single interventions by enhancing the impact of these interactions. Further research is required to determine the most effective combinations and strategies for individual patients.

## 7. Conclusion and prospects

Evidence highlighting a potential connection between gut microbiota and circadian rhythms is increasing. The gut microbiota and its metabolites can influence circadian rhythms through various mechanisms, including microbial oscillations, neurotransmitter production, vagus nerve signalling, epigenetic modifications and immune regulation. Conversely, the circadian system can affect the gut microbiota through circadian gene expression, neurotransmitters, vagus nerve signalling and immune responses. This bidirectional interaction provides insights into the pathogenesis of depression and can be referred to identify new therapeutic avenues. However, further research is needed to explore additional pathways linking the gut microbiota and circadian rhythms. Furthermore, direct evidence linking these interactions with depression is limited. The advancement of multi-omics technologies may offer further opportunities for both clinical and preclinical studies to gain a deeper understanding of this complex relationship.

## Data Availability

No due data was generated during the study.
